# Development of MetaXplore: An Interactive Tool for Targeted Metagenomic Analysis

**DOI:** 10.3390/cimb46050289

**Published:** 2024-05-15

**Authors:** Naima Bel Mokhtar, Elias Asimakis, Ioannis Galiatsatos, Amal Maurady, Panagiota Stathopoulou, George Tsiamis

**Affiliations:** 1 Laboratory of Systems Microbiology and Applied Genomics, Department of Sustainable Agriculture, University of Patras, 30100 Agrinio, Greece; naima1503@gmail.com (N.B.M.); eliasasim@gmail.com (E.A.); jgalia96@gmail.com (I.G.); panstath@upatras.gr (P.S.); 2 Laboratory of Innovative Technologies, National School of Applied Sciences of Tangier, Abdelmalek Essaâdi University, Tanger 93000, Morocco; amal.maurady.ma@gmail.com

**Keywords:** microbial profiling, shiny framework, amplicon data analysis, data visualization, taxonomic composition, diversity analysis, differential abundance

## Abstract

Over the last decades, the analysis of complex microbial communities by high-throughput sequencing of marker gene amplicons has become routine work for many research groups. However, the main challenges faced by scientists who want to make use of the generated sequencing datasets are the lack of expertise to select a suitable pipeline and the need for bioinformatics or programming skills to apply it. Here, we present MetaXplore, an interactive, user-friendly platform that enables the discovery and visualization of amplicon sequencing data. Currently, it provides a set of well-documented choices for downstream analysis, including alpha and beta diversity analysis, taxonomic composition, differential abundance analysis, identification of the core microbiome within a population, and biomarker analysis. These features are presented in a user-friendly format that facilitates easy customization and the generation of publication-quality graphics. MetaXplore is implemented entirely in the R language using the Shiny framework. It can be easily used locally on any system with R installed, including Windows, Mac OS, and most Linux distributions, or remotely via a web server without bioinformatic expertise. It can also be used as a framework for advanced users who can modify and expand the tool.

## 1. Introduction

The targeted metagenomic approach has been widely used to investigate the microbiota between different environmental or individual factors (e.g., treatments, geographical locations, diets, etc.). Analyzing the changes in composition and abundance of microbial communities provides valuable insight into ecosystem dynamics, including health, stability, and resilient changes, along with the ability to identify biomarkers that can serve as indicators for monitoring environmental changes, treatment responses, or disease [1]. Targeted sequencing is typically conducted based on conserved regions containing phylogenetically informative polymorphisms, such as the 16S rRNA gene for prokaryotes and the 18S rRNA or ITS genes for eukaryotes, which offer a powerful and economical way to characterize the bacterial community in large numbers of samples with affordable techniques [2,3,4]. The microbiome analysis pipeline includes mainly the clustering of sequences into OTUs or ASVs (Operational Taxonomic Units or Amplicon Sequence Variants) followed by taxonomic classification of the representative sequences. For instance, OTUs are typically clustered based on a sequence similarity threshold, often ranging from 97% to 99% [5]. Sequences belonging to the same OTUs represent a putative taxonomic unit at a specific taxonomic level (e.g., genus or species). Key discriminatory criteria include sequence similarity, which determines OTU membership using algorithms such as UPRASE [5] and CD-HIT [6], and taxonomic assignment, which assigns taxonomic lineage to OTUs based on reference databases such as Greengenes [7] or SILVA [8]. Clustering could also be influenced by sequencing errors and bias. These criteria are dynamic and evolve with sequencing technologies and bioinformatics tools. Various pipelines are commonly used for these tasks, including Mothur [9], QIIME [10], and USEACH [11]. However, despite their widespread use, they still have obvious shortcomings, such as a lack of efficient downstream statistical analyses and visualization solutions. The primary downstream analysis includes diversity analysis, taxonomic composition, differential abundance, biomarker identification, and core microbiome selection.

As the demand for comprehensive microbial analyses continues to grow, researchers have had to choose among a range of multipurpose software platforms and a wide range of analysis methods. For non-expert users, this task can be particularly intimidating, requiring a steep learning curve to effectively use the full potential of these tools. Furthermore, the increasing complexity of study designs, often involving multiple experimental conditions, necessitates the use of flexible analysis tools capable of dynamically adjusting analyses and visualizations in real time.

In the current study, we took advantage of the flexibility of R programming and the Shiny package to develop MetaXplore, an interactive, user-friendly platform that enables the discovery and visualization of microbial sequencing data. MetaXplore features an analytical pipeline for the analysis of microbial diversity and composition based on Operational Taxonomic Unit (OTU) tables. It is designed to provide microbiologists who are not skilled in programming with the ability to utilize R functionalities and conduct exploratory analyses of microbial sequencing data within a flexible and interactive GUI. In order to elucidate the functionalities of MetaXplore, we used a set of amplicon data representing the bacteria associated with the *Glossina morsitans morsitans* (*Gmm*) species. The example analysis aims to investigate the impact of a combination of supplements and antibiotics on the gut microbiota of mass-reared *Glossina* samples.

## 2. Methods and Materials

MetaXplore has an interactive, user-friendly interface written entirely in the R language using the Shiny framework, which is easy to use and does not require any programming skills. Like any Shiny interface, MetaXplore can be further customized or extended using HTML, CSS, and JavaScript. The current version is dependent on previously published packages, including GUnifrac [12] and APE [13] for distance calculations, vegan [14] for ordination methods, and ggplot2 [15] for visualization of results. MetaXplore is configured primarily for microbial ecology profiling using 16S rRNA gene, 18S rRNA and ITS sequencing data and requires four different files as input: (a) OTUs tables, which details the occurrence and abundance of each OTU within each sample, with taxa in rows and samples in columns, (b) a taxonomy file, a table containing the assigned taxonomy for each OTU, (c) Mapping file, containing experimental metadata in columns with a main column labeled “sample ID”, which contains sample labels matching those used in the OTU file, and at least one more column, (d) Tree file, a phylogenetic tree saved in Newick (NWK) format. Various unique or combined such as USEACH [11], QIIME [10] pipelines can be used to obtain these files. An example pipeline using both of these tools is illustrated in Figure S1.

### 2.1. Operation

MetaXplore is currently available in a web-based format (http://MetaXplore.eu; accessed on 12 May 2024) and can be accessed remotely from any web browser. It can also be launched locally from a computer running Linux, Windows, or MacOS with an R installation (version 4.1.2 or higher) and will run on any web browser. As MetaXplore auto-installs all required R-packages, no additional software requirements are needed. The source code of MetaXplore is available at https://github.com/nbel15/MetaXplore (accessed on 12 May 2024). Local installation instructions are also available. All interfaces and plots of MetaXplore are highly interactive, allowing users to visualize and download data in real time as well as to interact efficiently with the data and plots.

### 2.2. MetaXplore Sections

The MetaXplore interface provides different downstream analyses, classified in the following sections: (1) Import Data section to upload the dataset at the start of the analysis. (2) Alpha Diversity section to calculate alpha diversity indices among samples and visualize indices in boxplots with significant differences between the groups. (3) Beta Diversity section to calculate the beta diversity between samples and visualize groupings of samples according to a given category. (4) Relative Abundance section to calculate relative abundance matrices and produce heatmaps, stacked or standard bar charts for different taxonomy levels according to a given category. (5) Differential abundance section to test the significant variation in OTU abundance at different taxonomic levels. (6) Core microbiome section to identify the core OTUs based on the percentage of occurrence in samples and their abundance. And (7) Biomarker discovery section to identify the discriminant taxa between groups of samples.

#### 2.2.1. Data Import and Overview

Upon uploading the input data, MetaXplore will verify the format and validity of the input files. The OTU table, metadata, and taxonomy files can be saved either in TXT or TSV format, along with the OTU phylogenetic tree file in NWK format. It will identify the column in the metadata file labeled “sample ID”, match its contents with the header row in the OTU table and synchronize both files. The uploaded files are shown in different panels for easy access to the raw data.

#### 2.2.2. Alpha Diversity

Alpha diversity, which refers to the mean diversity of species within a sample or an experimental condition, is considered a ubiquitous approach in microbiome analysis surveys [16]. Different measures exist to estimate diversity. These measures reflect species richness, which is the number of species (OTUs) present, and species evenness, which is the equitability in the distribution of OTUs within a particular sample or a given category [17]. However, species diversity involves both the richness and evenness of individuals. To capture the structure of the community rather than enumerating the parts only, the two most popular indices are used, the Simpson and Shannon indices [18]. The Simpson index represents the probability that two randomly selected samples belong to the same species, which gives more weight to the evenness, meaning that rare species have a minimal impact on community diversity. In contrast, the Shannon index puts more weight on richness. The index is derived from information theory and represents the uncertainty of identifying the species that one randomly selected individual from the community will belong to, i.e., the more species in the community, the higher the uncertainty [19,20]. Another aspect of diversity is the phylogenetic diversity (Faith’s Phylogenetic Diversity; PD). PD quantifies the total branch lengths between the observed species on a rooted phylogenetic tree [21]. This metric provides a comparable evolutionary measure of biodiversity that cannot be captured by the species’ richness and/or their respective abundance [21,22,23]. MetaXplore allows users to select experimental categories and alpha diversity parameters simultaneously. Currently, MetaXplore supports several options of alpha diversity indices, including Shannon and Simpson, in addition to their effective values, richness indices such as ACE and Chao1, Evenness, and phylogenetic diversity (PD). The graphical results are presented in boxplots with experimental groups on the x-axis and indices on the y-axis annotated with significance labels (bars with asterisks) above the boxplots. Statistical comparisons of the indices between the groups are calculated using analysis of variance (ANOVA), followed by Tukey’s honestly significant difference (HSD).

#### 2.2.3. Beta Diversity

Beta diversity quantifies the distances between different microbial profiles described by the OTU table, which allows linking the overall taxonomic diversity pattern to the experimental features [24,25]. The most common approaches to calculate the similarity of microbial profiles are the Bray–Curtis dissimilarity index and the weighted and unweighted UniFrac distances. Quantitative metrics, including Bray–Curtis and weighted UniFrac, use OTU abundance data in calculations, whereas qualitative metrics, such as unweighted UniFrac, only consider the presence or absence. Unweighted and weighted UniFrac methods are sensitive to rare and dominant OTUs, respectively. To address this issue, a balanced alternative, known as Generalized UniFrac, was proposed [12]. Phylogenetic-based methods, such as UniFrac, exploit the similarities and differences among species, which generally provide an interpretable biological pattern [25,26]. Currently, MetaXplore allows users to choose between Bray–Curtis, weighted and unweighted UniFrac, and generalized UniFrac methods for the calculation of distance matrices. For visualizing the beta diversity analysis results in two-dimensional spaces, MetaXplore provides a variety of ordination methods, including Principal Coordinate Analysis (PCoA), Multi-Dimensional Scaling (MDS), its more robust and unconstrained non-metric version (NMDS), and Canonical Analysis of Principal Coordinate (CAP). The non-parametric permutation test PERMANOVA is used to assess the significant clustering between the groups [27]. In the case where the chosen data category contains more than two experimental groups, a pairwise comparison of the beta diversity metric is conducted, and the results are plotted in a heatmap graph.

#### 2.2.4. Relative Abundance

MetaXplore calculates the relative abundance with standard error of individual taxa within the selected experimental category. Users select a category from the metadata list, the taxonomic level(s) where all the OTUs with the same level will be combined, and the threshold of relative abundance taxon to visualize. The results can be displayed in various plots: stacked bar chart, heatmap, or basic bar chart. Additionally, users can download the average relative abundance along with the standard error table as a CSV file. All the plots can be customized in real-time by widgets, allowing the adjustment of label size, rotation of x-axis labels, graph colors, etc.

#### 2.2.5. Differential Abundance

The differential abundance analysis allows a systematic identification of the significant variation in OTUs between the assessed experimental conditions [28]. The statistical test is performed using nonparametric Kruskal–Wallis Rank Sum tests (for group > 2) followed by Wilcoxon Rank-Sum tests for each paired group as implemented by the microeco R package [29]. To avoid comparisons of taxa that may not be relevant in the experimental condition of interest (e.g., taxa with low relative abundance), MetaXplore offers the ability to identify mean abundance thresholds that can be set to filter out taxa with low abundance, as the pre-filtering of the data has been found to enhance the analytical power [30,31].

#### 2.2.6. Core Microbiome

The core microbiomes are typically quantified by the occurrence and abundance of microbial taxa across multiple samples [32]. These metrics are estimated from the standard OTU table. MetaXplore offers the ability to set a minimum abundance threshold, under which the taxon is disqualified from being part of the core microbiota. Then, the remaining taxa are evaluated based on their occurrence across samples using an assigned cutoff value. Overall, the minimum abundance threshold for OTUs to be considered for core membership typically ranges from 0.001% to 4.5%, while the occurrence cutoff ranges from 50% to 100% across different sample categories (e.g., geographical locations, host species, experimental treatments, etc.) [33,34]. Results are then presented in a presence–absence table for each qualified taxon, along with a Venn diagram comparing the different sample categories.

#### 2.2.7. Biomarker Discovery

The biomarker discovery analysis allows for the identification of specific taxa (also known as discriminant taxa) that exhibit characteristic differences between two or more experimental factors. This feature allows for the identification of potential indicators of diseases, different treatments, environmental changes, etc. The identification of discriminant taxa in MetaXplore is based on the LefSe algorithm [35] as implemented by the MicrobiotaProcess R package [36]. The identification is based on two steps: initially, identification of taxa with significant differences in relation to the selected factor using non-parametric Kruskal–Wallis tests. Then, the significant taxa are assessed by Linear Discriminant Analysis (LDA) to estimate the effect size of each taxon. The resulting output consists of a taxonomic tree listing all the taxa along with a bar chart highlighting the discriminative taxa according to the logarithmic LDA score (Effect Size) threshold (2.0 as default).

## 3. Results

As a use case of MetaXplore, we describe hereafter the main features of the platform and illustrate their utility via a microbiome study examining the dynamic of the bacterial community associated with laboratory-reared *Glossina morsitans morsitans* (*Gmm*) under different diets, with or without antibiotic treatment.

### 3.1. Dataset

The bacterial profiles were studied using amplicon sequencing of the hypervariable V3-V4 region of the 16S rRNA gene on gastrointestinal tract samples extracted from adult flies that were fed on diets enriched with three different supplements: B vitamins (Bvit), Vanderzant vitamin mixture (Van) and Yeast, with/without treatment with the antibiotic tetracycline (treated samples are labeled T/Bvit, T/Van and T/Yeast, respectively). Sample collection, dissection, DNA extraction, and sequencing were performed as described in [37]. Briefly, adult flies from both treated and untreated diets were surface sterilized with 70% ethanol and sterile PBS. DNA extraction from a pool of five guts for each sample was performed using a modified CTAB protocol [38]. The variable V3–V4 region of the bacterial 16S rRNA sequences was amplified using fusion primers U341F-MiSeq and 805R-MiSeq. High-throughput sequencing was performed by Macrogen using a 2 × 300 bp pair-end kit on a MiSeq platform. Sequencing resulted in a total of 850,135 reads. The NCBI Bioproject accession number for the raw sequencing data reported in this study is PRJNA1088284. Analysis of raw reads was conducted as previously described by using a combination of USEARCH and QIIME pipeline [37]. 

First, the OTU abundance table, taxonomy, and metadata files were loaded on the “Input Data section”. Uploading the phylogenetic tree is generally optional; it is only required if the user intends to perform a phylogenetically based analysis for alpha diversity (PD analysis) or beta diversity analysis that is based on the UniFrac distance matrix. After hitting the “Read Input” button, MetaXplore creates a phyloseq object [39] displaying the common samples and/or OTUs between the loaded files. This allows the user to reduce the number of samples and OTUs to include in the analysis by modifying only the metadata file and the taxonomy file, respectively.

### 3.2. Alpha Diversity Changes in Response to Diet

**Figure 1:**
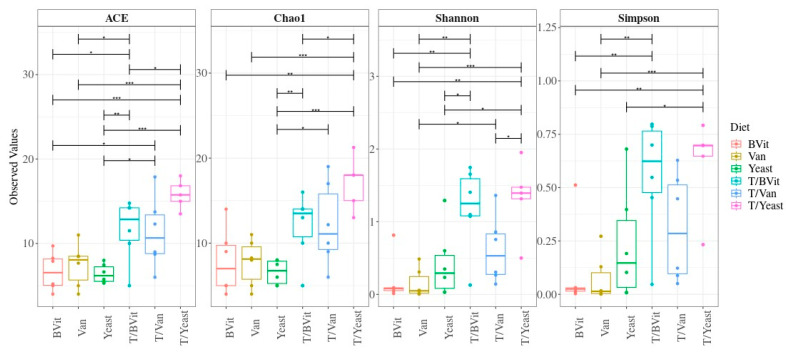
Species richness (ACE and Chao1) and diversity (Shannon and Simpson) within the examined diets. Diet supplemented with B vitamins (Bvit), Vanderzant vitamin mixture (Van), yeast, and their corresponding treated samples T/Bvit, T/Van, and T/Yeast, respectively. * *p* < 0.05, ** *p* < 0.01 and *** *p* < 0.001.

The bacterial community within the untreated *Gmm* samples fed on the supplement had a significantly lower species richness (Chao and ACE) and diversity (Shannon and Simpson) compared to their treated counterpart (Figure 1). Among the treated samples, *Gmm* samples supplemented with yeast showed the highest species richness and diversity compared to those supplemented with B vitamins (Bvit) and Vanderzant vitamin mixture (Van) (Figure 1).

### 3.3. Dynamic in Bacterial Diversity among Diets and Gender

**Figure 2:**
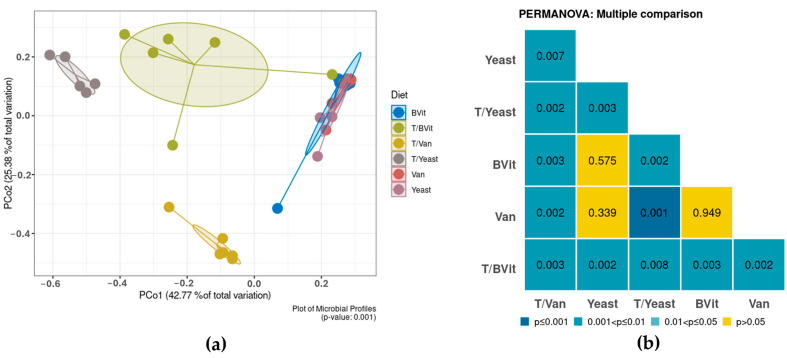
Bacterial diversity between the examined diets based on the GUnifrac dissimilarity matrix. (**a**) Principal Coordinates Analysis (PCoA). (**b**) PERMANOVA pairwise comparison between diets.

Different bacterial diversity patterns were observed among communities fed on varying diets (Figure 2). Untreated *Gmm* flies fed on diets enriched with supplements form a tight cluster compared to their treated counterparts, which form separate clusters (Figure 2a). The PERMANOVA test indicated that no significant difference was detected between the untreated diets, which indicates that, to some extent, the type of supplement did not alter the bacterial community of the host (*p*-value > 0.05; Figure 2b). In contrast, significantly different bacterial communities were observed between the tetracycline-treated samples fed on different supplements (*p*-value < 0.05; Figure 2b). On the other hand, no significant difference was observed between males and females (*p*-values = 0.471, Figure S2).

### 3.4. Taxonomic Composition in Relation to Rearing Diet

**Figure 3:**
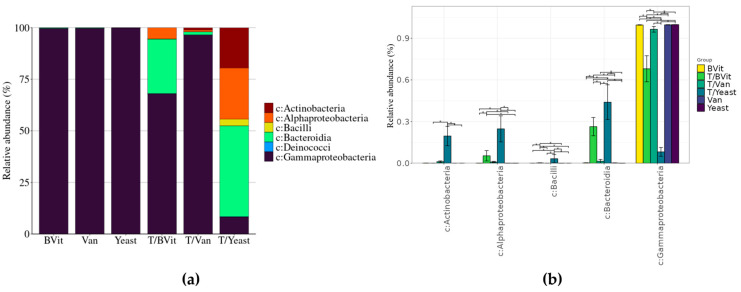
Composition of bacterial community associated with *Gmm* gut according to diets. (**a**) Relative abundance of taxa at Class level. (**b**) Differentially abundant classes. * *p* < 0.05.

Different bacterial compositions were observed between the treated and untreated samples (Figure 3). At the class level, the guts of untreated *Gmm* samples fed on diets enriched with supplements were dominated by members of Gammaproteobacteria, representing more than 99% of the bacterial communities (Figure 3a). Treated samples supplemented with Vanderzant vitamin mixture maintain the dominance of members of Gammaproteobacteria (96.4 ± 2.1%) with a slight increase in members of Bacteroidia, Actinobacteria, and Alphaproteobacteria. In the samples supplemented with B vitamins, tetracycline treatment significantly reduced the presence of Gammaproteobacteria (68 ± 9.4%), leading to a significant increase in members of Bacteroidia and Alphaproteobacria (26.4 ± 6.5% and 5.4 ± 3.7%, respectively) (Figure 3a,b). The samples supplemented with yeast showed the highest influence with the tetracycline treatment, where Gammaproteobacteria significantly decreased to (8.2 ± 2.3%) along with a significant increase in members of Bacteroidia (43.9 ± 11.9%), Alphaproteobacteria (24.7 ± 8.5%), Actinobacteria (19.6 ± 6.3%), and Bacilli (3.3 ± 2.7) (Figure 3a,b).

**Figure 4:**
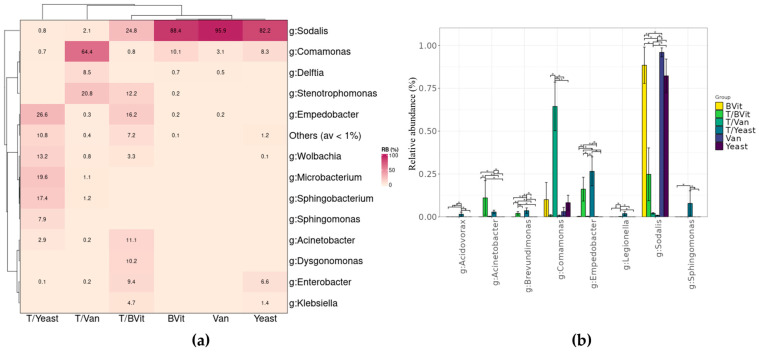
Composition of bacterial community associated with *Gmm* gut according to diets. (**a**) Relative abundance of taxa at the Genus level. (**b**) Differentially abundance genera. * *p* < 0.05.

At the Genus level, the gut bacterial communities of the untreated samples were mainly dominated by *Sodalis,* representing 88.4 ± 10.5%, 95.9 ± 2.6%, and 82.2 ± 9.8% in *Gmm* samples fed on a diet enriched with B vitamins, Vanderzant vitamin mixture and yeast, respectively (Figure 4a). Interestingly, *Wigglesworthia,* which is an obligatory symbiont of *Glossina* flies [40,41,42] was under the detection level. This indicates that the supplements assessed in this study prevent the proliferation of these bacteria. The relative abundance of *Sodalis* was significantly decreased in tetracycline-treated samples regardless of the supplement used (Figure 4a,b). On the other hand, *Empedobacter* was significantly increased in the treated samples that were fed on a diet supplemented with B vitamins and yeast (16.2 ± 6.9% and 26.6 ± 7.7%, respectively). Similarly, Vanderzant vitamin mixture supplements in the treated samples enhanced the presence of *Comamonas* (64.4 ± 14.2%) with the tetracycline treatments (Figure 4a,b).

### 3.5. Core Microbiome among Treated and Untreated Samples

**Figure 5:**
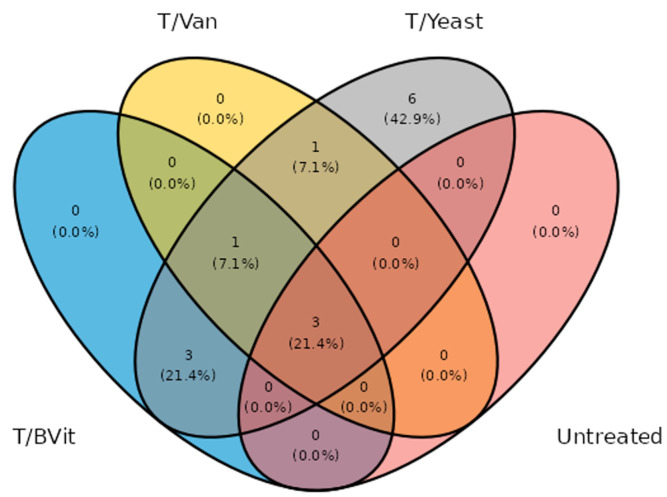
Venn diagram of the distribution of core taxa determined in *Gmm* gut under the assessed diets, with a threshold set at 75%.

At 75% prevalence and 0.01% abundance thresholds, a total of three OTUs were identified as the core bacteriome of *Gmm* samples (*Sodalis*, *Empedobacter,* and *Wolbachia*), together representing around 60% of the total bacterial community (Figure 5 and Table 1). *Comamonas* was detected as a core microbiome member in all the tetracycline-treated samples, while *Acinetobacter*, *Bacillus,* and *Brevundimonas* were detected in treated samples fed on a diet enriched with B vitamins and yeast supplement. Six core OTUs were detected in treated samples with yeast supplements that are not shared with the other diets, and these include *Microbacterium*, *Sphingomonas*, *Acidovorax*, *Cupriavidus*, *Acinetobacter,* and *Meiothermus* (Figure 5).

### 3.6. Discriminant Taxa between Treated and Untreated Samples

**Figure 6:**
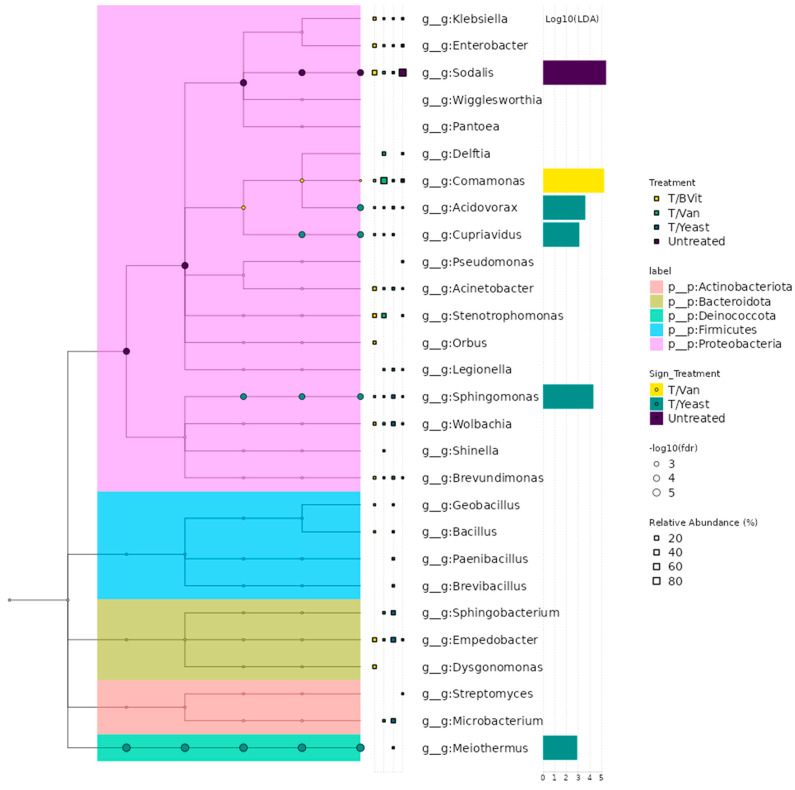
Discriminant taxa between treated and untreated diets at the Genus level. The tree represents the phylogenetic relationships of the identified genera. The horizontal bars represent the logarithmic LDA score of the discriminant genera.

Based on the LEfSe approach, the untreated samples were significantly characterized by the *Sodalis* genus, treated samples fed on Vanderzant vitamin mixture with *Comamomas,* and those fed on yeast were characterized by various genera, including *Sphingomonas*. In contrast, no discriminative taxon was found for the treated samples supplemented with B vitamins (Figure 6).

## 4. Discussion and Conclusions

The generation of amplicon sequencing data to explore microbial communities has become routine in numerous research laboratories, using technologies such as Illumina and Oxford Nanopore. Research institutes and scientists are consistently focusing on the adoption of open-source software for the analysis of these data. R programming environment stands out as the most used tool, offering a free environment for statistical computing, flexible and continuously supported by a large community of active users, developers, and researchers. The development of the shiny and shin-dashboard packages facilitated the development of web applications within the R framework. Consequently, MetaXplore has been implemented using both tools along with functions from various R packages and statistical programs. MetaXplore was created to provide dynamic experience for rapid data analysis of complex microbial communities generated by high-throughput sequencing of community markers such as the 16S and 18 rRNA genes or the ITS region. The current release provides the fundamental downstream analyses required for publications and is rooted in the most up-to-date methods used in the field of microbial ecology. The use of the R language for the development of the entire platform makes MetaXplore a suitable framework for advanced users who can modify and expand the tool.

## Figures and Tables

**Table 1:** Core bacterial taxa determined in *Gmm* gut under the assessed diets, with a threshold set at 75%.

OTUs	Phylum	Class	Genus	T/BVit	T/Van	T/Yeast	Untreated
Otu1	p:Proteobacteria	c:Gammaproteobacteria	*g:Sodalis*	1	1	1	1
Otu2	p:Bacteroidota	c:Bacteroidia	*g:Empedobacter*	1	1	1	1
Otu6	p:Proteobacteria	c:Alphaproteobacteria	*g:Wolbachia*	1	1	1	1
Otu7	p:Proteobacteria	c:Gammaproteobacteria	*g:Comamonas*	1	1	1	0
Otu11	p:Proteobacteria	c:Gammaproteobacteria	*g:Acinetobacter*	1	0	1	0
Otu15	p:Firmicutes	c:Bacilli	*g:Bacillus*	1	0	1	0
Otu3	p:Proteobacteria	c:Alphaproteobacteria	*g:Brevundimonas*	1	0	1	0
Otu4	p:Proteobacteria	c:Gammaproteobacteria	*g:Legionella*	0	1	1	0
Otu19	p:Actinobacteriota	c:Actinobacteria	*g:Microbacterium*	0	0	1	0
Otu22	p:Proteobacteria	c:Alphaproteobacteria	*g:Sphingomonas*	0	0	1	0
Otu23	p:Proteobacteria	c:Gammaproteobacteria	*g:Acidovorax*	0	0	1	0
Otu26	p:Proteobacteria	c:Gammaproteobacteria	*g:Cupriavidus*	0	0	1	0
Otu27	p:Deinococcota	c:Deinococci	*g:Meiothermus*	0	0	1	0
Otu991	p:Proteobacteria	c:Gammaproteobacteria	*g:Acinetobacter*	0	0	1	0

## Data Availability

MetaXplore is available at http://metaxplore.eu website (accessed on 12 May 2024). The complete source code is available at https://github.com/nbel15/MetaXplore (accessed on 12 May 2024). Raw sequencing data reported in this study are available under the NCBI accession number PRJNA1088284.

## Supplementary Materials

The following supporting information can be downloaded at https://www.mdpi.com/article/10.3390/cimb46050289/s1. Figure S1: Exemplar pipeline to generate MetaXplore input files, Figure S2: Principal Coordinates Analysis (PCoA) of bacterial diversity between males and females.
