# Supplementary material for: Development of MetaXplore: An Interactive Tool for Targeted Metagenomic Analysis
Source: Curr Issues Mol Biol. 2024 May 15;46(5):4803–14. doi: 10.3390/cimb46050289 (PMC11120546; doi:10.3390/cimb46050289)
Supplement: Supplementary file 1 [file cimb-46-00289-s001.zip › cimb-2989283-supplementary.pdf]

Supplementary Materials

Development of MetaXplore: An Interactive Tool for targeted metagenomic Analysis

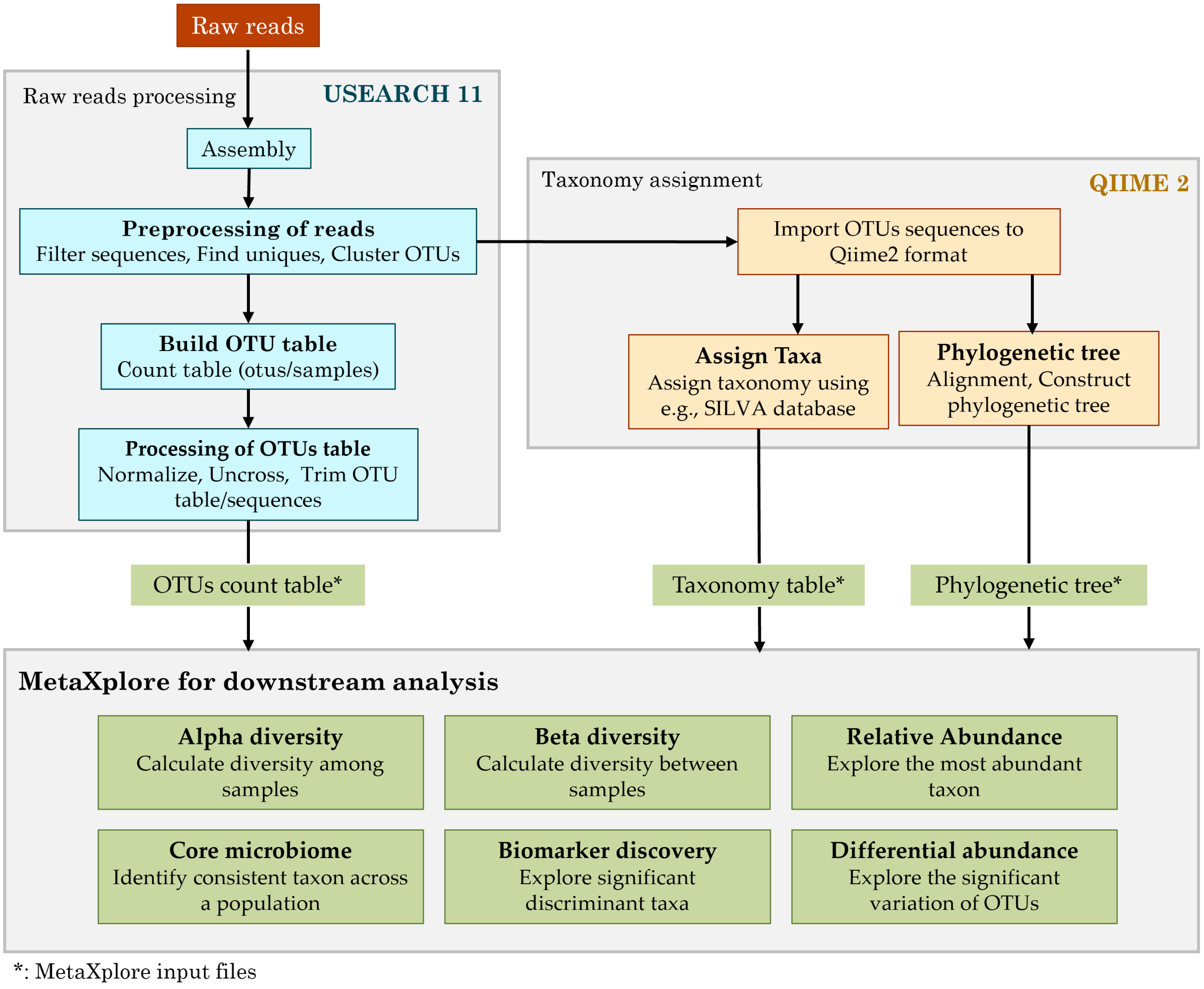

Figure S1: Exemplar pipeline to generate MetaXplore input files.

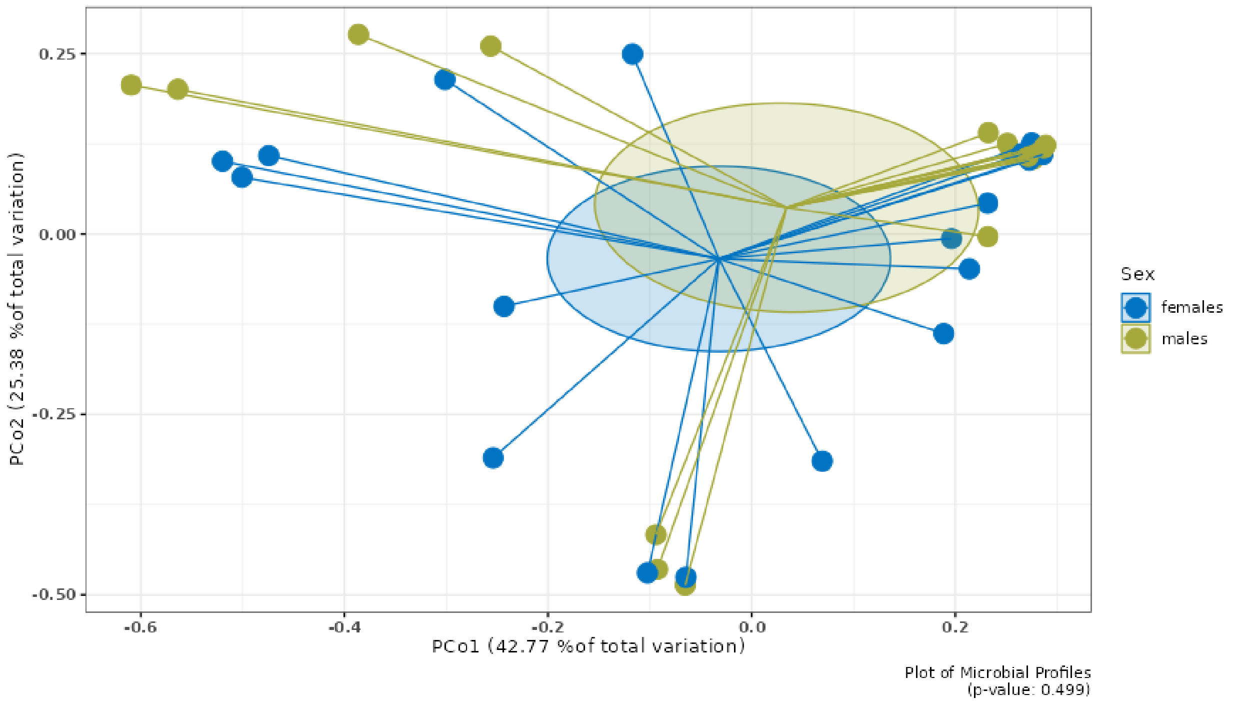

Figure S2: Principal Coordinates Analysis (PCoA) of bacterial diversity between males and females.
